# Icariin Attenuates M1 Activation of Microglia and Aβ Plaque Accumulation in the Hippocampus and Prefrontal Cortex by Up-Regulating PPARγ in Restraint/Isolation-Stressed APP/PS1 Mice

**DOI:** 10.3389/fnins.2019.00291

**Published:** 2019-03-28

**Authors:** Yihe Wang, Tianrui Zhu, Min Wang, Feng Zhang, Guitao Zhang, Jing Zhao, Yuanyuan Zhang, Erxi Wu, Xiaohong Li

**Affiliations:** ^1^School of Medicine, Shandong University, Jinan, China; ^2^Department of Neurology, Jinan Central Hospital, Shandong University, Jinan, China; ^3^Department of Neurosurgery and Neuroscience Institute, Baylor Scott & White Health, Temple, TX, United States; ^4^Department of Surgery and Department of Pharmaceutical Sciences, Texas A&M University Health Science Center, College Station, TX, United States; ^5^LIVESTRONG Cancer Institutes, Dell Medical School, The University of Texas at Austin, Austin, TX, United States

**Keywords:** stress, Alzheimer’s disease, microglia, icariin, PPARγ, cytokine, hippocampus, prefrontal cortex

## Abstract

**Background:**

Studies have shown that psychosocial stress is involved in Alzheimer’s disease (AD) pathogenesis; it induces M1 microglia polarization and production of pro-inflammatory cytokines, leading to neurotoxic outcomes and decreased β-amyloid (Aβ) clearance. Icariin has been proven to be an effective anti-inflammatory agent and to activate peroxisome proliferator-activated receptors gamma (PPARγ) which induces the M2 phenotype in the microglia. However, whether restraint/isolation stress reduces the clearance ability of microglia by priming and polarizing microglia to the M1 phenotype, and the effects of icariin in attenuating the inflammatory response and relieving the pathological changes of AD are still unclear.

**Methods:**

APP/PS1 mice (male, aged 3 months) were randomly divided into a control group, a restraint/isolation stress group, and a restraint/isolation stress + icariin group. The restraint/isolation stress group was subjected to a paradigm to build a depressive animal model. Sucrose preference, open field, elevated plus maze, and Y maze test were used to assess the stress paradigm. The Morris water maze test was performed to evaluate spatial reference learning and memory. Enzyme-linked immunosorbent assay and immunohistochemistry were used to identify the microglia phenotype and Aβ accumulation. Western blotting was used to detect the expression of PPARγ in the hippocampus and prefrontal cortex (PFC).

**Results:**

Restraint/isolation stress induced significant depressive-like behaviors in APP/PS1 mice at 4 months of age and memory impairment at 10 months of age, while 6 months of icariin administration relieved the memory damage. Restraint/isolation stressed mice had elevated pro-inflammatory cytokines, decreased anti-inflammatory cytokines, increased Aβ plaque accumulation and more M1 phenotype microglia in the hippocampus and PFC at 10 months of age, while 6 months of icariin administration relieved these changes. Moreover, restraint/isolation stressed mice had down-regulated PPARγ expression in the hippocampus and PFC at 10 months of age, while 6 months of icariin administration reversed the alteration, especially in the hippocampus.

**Conclusion:**

Restraint/isolation stress induced depressive-like behaviors and spatial memory damage, over-expression of M1 microglia markers and more severe Aβ accumulation by suppressing PPARγ in APP/PS1 mice. Icariin can be considered a new treatment option as it induces the switch of the microglia phenotype by activating PPARγ.

## Introduction

Alzheimer’s disease (AD) is a neurodegenerative disorder characterized by progressive cognitive impairment (Selkoe and Schenk, 2003). Prominent neuropathological features of AD are β-amyloid (Aβ) plaques and neurofibrillary tauopathy consisting of threads and tangles (NFT), which are observed throughout the brain, including the areas critically involved in memory formation and emotional regulation (Braak and Braak, 1996).

Mounting evidence shows that psychosocial stress is involved in AD pathogenesis, as patients with posttraumatic stress disorder (PTSD) or depression often develop dementia and even clinical AD (Green et al., 2003; Wilson et al., 2005; Qureshi et al., 2010; Yaffe et al., 2010; Gracia-Garcia et al., 2015; Sacuiu et al., 2016). The elevated level of cortisol, a stress hormone, found in AD patients plays a crucial role in contribution to the comorbidity (Hartmann et al., 1997; Elgh et al., 2006). Studies in animal models further demonstrated the association of hypothalamic–pituitary–adrenal axis activation with AD pathogenesis in the frontal cortex and hippocampus (Green et al., 2006; Sotiropoulos et al., 2008; Joshi et al., 2012; Justice et al., 2015). Chronic unpredictable stress (CUS) significantly increased both serum corticosterone levels (Liu et al., 2010; Yang et al., 2012) and amyloid precursor protein (APP) fragments. Aβ infusion triggered APP misprocessing in the hippocampus and prefrontal cortex (PFC) of rats, which was further exacerbated by stress (Catania et al., 2009). Furthermore, chronic stress including restraint/isolation stress in particularly elevated Aβ40 and Aβ42 levels of brain, accelerated amyloid plaque formation and impaired learning and memory in mice (Jeong et al., 2006; Carroll et al., 2011; Lee and Han, 2013). More importantly, a recent study demonstrated that prenatal stress induced a constant neuroinflammatory response which contributes to a more vulnerable profile; the latter can subsequently lead to an aberrant response to accumulating Aβ peptides, and ultimately modify the extent of Aβ neuropathology. The study concluded that early-life stress aggravated plaque pathology in 10-month-old APP/PS1 mice, and was accompanied by reduced microglial accumulation and increased level of pro-inflammatory tumor necrosis factor-α (TNF-α) (Hoeijmakers et al., 2016).

Microglial activation is often classified into classical (M1) and alternative (M2). M1 microglia may contribute to dysfunction of the neurotrophic system by expressing pro-inflammatory cytokines, such as TNF-α, interleukin-1β (IL-1β) and IL-6 (Michelucci et al., 2009). In the M1 phenotype of the microglia, the activation of nuclear factor κB (NF-κB) may play a critical role in the production of pro-inflammatory cytokines, leading to neurotoxic outcomes (Park et al., 2015). The M2 phenotype, also known as the neuroprotective microglial phenotype, releases different mediators including IL-4, IL-10, and transforming growth factor-β (TGF-β) (Michelucci et al., 2009) to antagonize inflammation-induced damage in the central nervous system (CNS) (Kobayashi et al., 2013; Zhao et al., 2015), and is associated with enhanced phagocytosis of deposited amyloid (Mandrekar-Colucci et al., 2012; Heneka et al., 2013; He et al., 2016; Huang et al., 2017). Stress paradigms, including CUS and chronic social defeat stress, are among the factors that could induce M1 microglia polarization (Zhao et al., 2016; Tang et al., 2018).

Peroxisome proliferator-activated receptors (PPARs) are a group of nuclear receptor proteins regulating gene expression as ligand-activated transcription factors (Michalik et al., 2006). Three closely related PPAR isoforms have been identified (alpha, delta, and gamma), transcribed from different genes and characterized by different tissue distribution, ligand specificity, and physiological roles (Berger and Moller, 2002; Breidert et al., 2002; Chang et al., 2007; Tontonoz and Spiegelman, 2008). Among the three isoforms, PPARγ has the highest expression in the CNS, where it has been identified in neurons, astrocytes, and glial cells (Moreno et al., 2004). Specifically, PPARγ appears to be linked to stress modulation (Garcia-Bueno et al., 2005) and to mediate the conversion between microglia phenotypes (Yamanaka et al., 2012; Saijo et al., 2013). PPARγ induced the M2 phenotype microglia under Aβ toxicity via adiponectin treatment through its effects on the anti-inflammatory response (Song et al., 2017). Moreover, pioglitazone which was used to treat depressive-like behaviors in a chronic mild stress mouse model is associated with PPARγ-mediated M2 activation of the microglia (Zhao et al., 2016).

Icariin, a natural flavonoid compound extracted from Epimedium brevicornum Maxim (a traditional Chinese herb), has been proven to have a wide range of effects including anti-tumor, anti-oxidant, anti-bacterial, and anti-inflammatory properties (Luo et al., 2007; Zhou et al., 2011). Previous studies investigated the pharmacological properties of icariin in the CNS and showed that it can markedly attenuate cognitive deficits in several models of AD (Li et al., 2010; Nie et al., 2010; Jin et al., 2014) and alleviate the neuronal injury induced by ischemia (Li et al., 2005). It was also reported that icariin can attenuate the inflammatory response through PPARγ activation in rats (Xiong et al., 2016).

Previous studies also showed that various stress can activate M1 microglia (Zhao et al., 2016; Tang et al., 2018), which is associated with decreased Aβ clearance. In the present study, we first hypothesized that restraint/isolation stress might prime and polarize microglia to the M1 phenotype and reduce its clearing ability. Second, we aimed to probe the effects of icariin in attenuating the inflammatory response and relieving the pathological changes of AD by targeting PPARγ activation in the hippocampus and PFC of APP/PS1 double-transgenic mice.

## Materials and Methods

### Animals and Experimental Procedure

**Figure 1:**
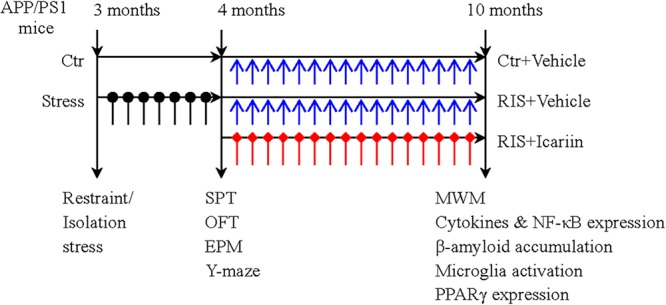
The experimental paradigm. Thirty male APP/PS1 mice were randomly divided into three groups (10 mice per group): control group (Ctr), restraint/isolation stress group (RIS), and restraint/isolation stress + icariin group (RIS+ICA). Mice in both the RIS and RIS+ICA groups were subjected to 28 days of restraint/isolation stress procedure, from 3 to 4 months of age. Mice in the RIS+ICA group were administered icariin (60 mg/kg) daily for 6 months, from 4 to 10 months of age. The weight of the mice was recorded at baseline, every week during the stress procedure and every month during the period of drug administration. The behavioral tests performed after stress included the sucrose preference test (SPT), open field test (OFT), elevated plus maze (EPM) and Y maze test in this order. After drug administration, the Morris water maze (MWM) test was performed. Enzyme-linked immunosorbent assay and immunohistochemistry were used to assess the microglia phenotype and western blotting was used to measure the expression of PPARγ in the hippocampus and PFC.

Thirty male APP/PS1 mice, aged 3 months and weighing 24–30 g, obtained from Beijing HFK Bioscience Co., Ltd. (Beijing, China), were housed (five per cage) and maintained under a 12-h light-dark cycle, at 20–24°C with free access to food and water. After 7 days of adaptation, the mice were randomly divided into three groups (10 mice per group): a control group, a restraint/isolation stress group (RIS), and a restraint/isolation stress + icariin group (RIS+ICA). Mice in both the RIS and RIS+ICA groups were subjected to 28 days of restraint/isolation stress from 3 to 4 months of age. The mice in the RIS+ICA group received daily administration of icariin (60 mg/kg) for 6 months from 4 to 10 months of age. The mice weight was recorded at baseline, every week during the stress procedure, and every month during drug administration. Behavioral tests after stress included the sucrose preference test (SPT), open field test (OFT), elevated plus maze test and Y maze test. The Morris water maze (MWM) test was used to measure learning and memory after drug administration at 10 months of age. Enzyme-linked immunosorbent assay (ELISA) and immunohistochemistry were used to detect Aβ plaque accumulation and microglia phenotypes, whereas western blotting was used to detect the expression of PPARγ in the hippocampus and the PFC. The experimental paradigm is presented in Figure 1.

### Restraint/Isolation Stress and Drug Treatments

Restraint/isolation stress is a paradigm used to build depressive animal models (Carroll et al., 2011). Each mouse was individually restrained in restraint tubes for 6 h per day, and the mice were deprived of water and food during the restraint/isolation stress.

Icariin (Cat no. I8760, Beijing Solarbio Science & Technology Co., Ltd.) was orally administered daily to the mice in the RIS+ICA group (60 mg/kg, freshly suspended in ddH_2_O) from 4 to 10 months of age (Wang et al., 2013; Li et al., 2015; Hoeijmakers et al., 2017). The mice in the control group and restraint/isolation stress group were given ddH_2_O (vehicle) in the same volume to balance the systematic error.

### Sucrose Preference Test (SPT)

The SPT test was carried out at the end of the exposure to restraint/isolation stress. A decreased sucrose preference is considered to be homologous to anhedonia, the inability to experience pleasure, and thus simulates the defining symptom of major depression. Mice were individually housed during the SPT. Before the test, the mice were trained to adapt to sucrose solution (1%, w/v): Two bottles of sucrose solution were placed in each cage for 24 h, then one bottle of sucrose solution was replaced with pure water for 24 h. After adaptation, mice were deprived of water and food for 24 h. Then, each mouse was given free access to two bottles for 1 h, one with 200 ml of 1% (w/v) sucrose solution and the other with 200 ml of pure water. The drinking bottles were weighed to calculate fluid consumption 1 h later. The sucrose preference percentage was evaluated as the amount of sucrose solution consumed during consumption of all fluids.

### Open Field Test (OFT)

The OFT was used to test the motivation of mice to explore. The open field apparatus is a white wooden box (90 cm in diameter and 45 cm in wall height). The bottom of the apparatus is divided into 25 squares. The mice were individually placed at the center of the apparatus and left free to explore the arena for 5 min. The following indices were recorded: number of grid crossings (horizontal movement), defined as crossing into the nearby grids with more than three paws or half of the body; and number of rears (vertical movement), defined as both forelimbs raised at least 1 cm above the ground. The box was thoroughly cleaned between tests. Horizontal and vertical movements were recorded using a camera linked with a computer fitted with a SMART video tracking system (SMART v3.0, Panlab, Spain).

### Elevated Plus Maze (EPM)

The experimental apparatus consisted of a central part (5 cm× 5 cm), two opposing open arms (30 cm × 5 cm) and two opposing closed arms (30 cm × 5 cm) with non-transparent walls of 15 cm height. The maze was elevated 50 cm above the floor. The mice were individually placed in the center of the maze facing an open arm and allowed free exploration for 5 min. The number of entries into the open and closed arms and the total time spent in the open and closed arms were recorded and measured by the SMART video tracking system. The time and entry ratios were used to obtain the anxiety score. The time ratio was defined as the ratio of total time spent in the open arms of the maze to the total time spent in any arm. The entry ratio was defined as the ratio of the number of entries into the open arms of the maze to the total number of entries into any arm of the maze. An anxiety score was calculated as 1 - (time ratio + entry ratio/2). Anxious mice were more likely to stay in the closed arms, so that a smaller time ratio or entry ratio indicates more anxious behavior. The maze platforms and walls were thoroughly cleaned with 75% ethanol between test sessions and allowed to dry.

### Y Maze Test

The Y maze test was used to assess various parameters related to spatial memory. The apparatus consisted of three arms (50 cm × 16 cm × 32 cm) made of black non-transparent plastic to form a “Y” shape. Visual cues made of colored paper were placed on the walls of the arms. The floor of the maze was covered with padding. The mice were placed into one of the arms of the maze (start arm) and allowed to explore the maze with one of the arms closed for 15 min (training trial). One hour later, the mice were returned to the start arm, and allowed to explore all three arms of the maze freely for 5 min (test trial). At the end of the training trial, the mice were returned to their home cage and the padding inside the maze was mixed to reduce the possibility of odor interference. The number of entries, the time spent and the distance traveled in each arm were measured by the SMART video tracking system. Ratio time was defined as the total time spent in the novel arm divided by the total time spent in any arm of the maze. Ratio entry was defined as the number of entries into the novel arm divided by the total number of entries into any arm of the maze. Ratio distance was defined as the total distance covered in the novel arm divided by the total distance covered in any arm of the maze. Due to their natural curiosity, mice are more likely to explore the novel arm, so that these parameters reflect spatial recognition memory. More entries or exploration of the novel arm indicate better spatial memory.

### Morris Water Maze (MWM)

The MWM test was performed to evaluate spatial reference learning and memory of APP/PS1 mice at 10 months old. The maze was a blue pool (0.8 m in diameter) filled with water (0.3 m deep, at 25 ± 1°C). Geometric pictures pasted on the surrounding walls were used by the mice for spatial orientation. The maze was divided into four equal quadrants corresponding to four directions: I, II, III, and IV. The mice movements were captured by a CCD camera connected with a computer. All mice were allowed to swim freely for 60 s within 24 h before the formal training. During the following 5 consecutive days, mice were trained to find a platform (12 cm in diameter) hidden under the water surface in quadrant IV four times per day. If a mouse failed to find the platform within 60 s, it was manually guided to the platform and allowed to remain there for 10 s, and the escape latency was scored as 60 s. On the sixth day, all mice were released into the maze, without the hidden platform, from an identical point of quadrant I and allowed to swim freely for 60 s. The escape latency of training days as well as the percentage of time spent in and the number of entries into quadrant IV on the sixth day were analyzed with the SMART video tracking system.

### Protein Isolation

Mice (six per group) were sacrificed under deep anesthesia, and both sides of the frontal and parietal bones were removed to obtain the whole brain from the cranial cavity. The PFC and the hippocampus were collected and immediately immersed in liquid nitrogen, and stored at -80°C for later protein isolation. The tissue was dissociated using an ultrasonic cell disruptor and lysed in a cold lysis buffer containing 10 mM Tris-HCl, pH 8.0, 240 mM NaCl, 5 mM EDTA, 1 mM dithiothreitol, 0.1 mM phenylmethanesulfonyl fluoride, 1% Triton X-100, 1 mM sodium vanadate and 1 g/ml of leupeptin, pepstatin and aprotinin. Tissue lysates were incubated at 4°C for 20 min. The samples were centrifuged at 12,000 rpm for 10 min at 4°C, then for each sample the supernatant was collected and protein content was determined using BCA protein assay reagents (Beyotime Institute of Biotechnology, China).

### Enzyme-Linked Immunosorbent Assay (ELISA)

The levels of IL-1β, IL-6, TNF-α, IL-4, IL-10, TGF-β1, NF-κB component p65, and Aβ 1-42 were measured using commercially available ELISA kits according to the manufacturer’s instructions (Beijing Andy Huatai Technology Co., Ltd., China). Briefly, serial dilutions of protein standards and samples were added to 96-well ELISA plates followed by HRP labeled antibodies for IL-1β, IL-6, TNF-α, IL-4, IL-10, TGF-β1, NF-κB p65, and Aβ 1-42 to form antibody-antigen-enzyme labeled antibody complexes. After complete washing with wash solution, TMB substrate solution was added, which under the catalysis of HRP was converted to blue. The reaction was stopped using the stop solution. Optical density at 450 nm was detected using the iMark Microplate Absorbance Reader (Bio-Rad, United States). The concentration of each sample was calculated from the linear equation derived from the standard curve of known concentrations of NF-κB p65, Aβ 1-42, and the cytokines.

### Western Blotting

Brain protein samples containing the same amount of total protein were mixed with a 5x Laemmli loading buffer (protein volume: loading buffer = 4: 1). The mixed protein sample was heated at 99°C for 5 min to achieve protein denaturation, then 15 μg of protein sample was separated on 10% sodium dodecyl sulfate-polyacrylamide (SDS-PAGE) gel and electro-transferred to polyvinylidene difluoride (PVDF) membranes (Servicebio, China). The membrane was blocked with 5% skim milk in TBS containing 0.1% Tween-20 (TBST) for 1.5 h and incubated in a refrigerator with primary antibodies against PPARγ (1:1000, Abcam, United States) or GAPDH (1:10000, Biogot Technology Co., Ltd., China) at 4°C overnight. The following day, after washing with TBST three times for 5 min, the PVDF membrane was incubated for 1 h with the secondary antibody (ZSGB-BIO, China). Then, the PVDF membrane was washed again with TBST three times for 15 min; the western blots were visualized after being incubated with ECL solution (Millipore Corp., United States) for 1 min and exposed onto photographic films (Eastman Kodak Company, United States) for 10–90 s. Signal intensities were quantified using the ImageJ 14.0 software, and the intensity value of the protein band of interest was normalized according to that of the GAPDH band of the same sample.

### Immunohistochemistry

The mice (four per group) were anesthetized with pentobarbital and perfused with 50 ml of 0.1 M PBS, followed by 100 ml of ice-cold 4% paraformaldehyde (PFA). Paraffin-embedded PFC and hippocampus tissues were cut into 5 μm thick sections with a microtome. Coronal sections of the hippocampus were selected between bregma -1.22 mm and bregma -3.64 mm, and sections of the PFC were selected between anterior +4.7 mm to bregma posterior +3.7 mm to bregma. After rehydration, the sections were heated at 95–98°C in 0.01 M citrate buffer (pH 6.0) for 15 min, then cooled to room temperature for 30 min and washed with PBS. Thereafter, the sections were incubated with fresh 3% H_2_O_2_ for 25 min at room temperature to block endogenous peroxidase activity. After washing with PBS, the sections were blocked with 3% BSA for 30 min, incubated with mouse anti-Aβ 1-42 antibody (1:1000, BioLegend, United States), rabbit anti-ionized calcium-binding adapter molecule 1 (Iba-1) antibody (1:2000, Servicebio, China) or rabbit anti-inducible nitric oxide synthase (iNOS) antibody (1:100, Abcam, United States) at 4°C overnight. After washing with PBS, the sections were incubated with HRP labeled anti-mouse or anti-rabbit antibodies at room temperature for 50 min, rewashed with PBS and incubated in diaminobenzidine (DAB) (Servicebio, China) for chromogen development under a microscope. Finally, the sections were dehydrated, cleared and mounted. After completing the staining, each section was viewed under 400× magnification. The expression of Aβ and the positive reactive cells of Iba-1 and iNOS were observed. Sections from corresponding locations were selected in each group, and five different visual fields (50 μm × 50 μm) of expression areas in the hippocampus [CA1, CA3, and dentate gyrus (DG)] and PFC were selected in each section under a high power lens to count Iba-1- and iNOS-positive cells.

### Statistics

Data are presented as mean ± standard error of mean (SEM). Statistical analysis was carried out by one-way analysis of variance (ANOVA) with Student–Newman–Keuls (SNK) *post hoc* test. Differences were considered statistically significant if the *p*-value was less than 0.05.

## Results

### Effect of RIS on Behavioral Changes in SPT, OFT, EPM, and Y Maze Test

#### Sucrose Preference Test

**Figure 2:**
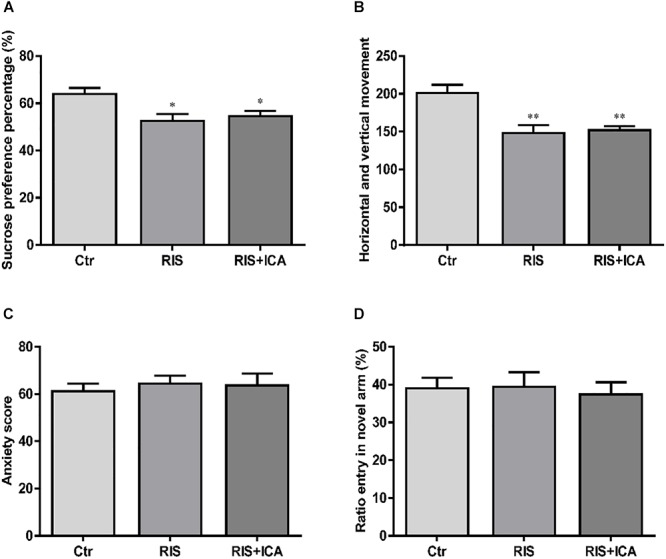
Effect of RIS on behavioral changes in SPT **(A)**, OFT **(B)**, EPM **(C)**, and Y-maze test **(D)**. **(A)** Stress exposure significantly reduced the percentage of sucrose consumption in stressed APP/PS1 mice, in both the RIS and the RIS+ICA groups, compared with the control animals. **(B)** APP/PS1 mice in the two stress groups showed decreased horizontal and vertical movements compared with the control group. **(C)** Four weeks of RI stress exposure did not significantly change the anxiety score of mice in the two stress groups. **(D)** Four weeks of RI stress exposure did not significantly reduce the ratio entry. The results are expressed as the mean ± SEM, *n* = 10. ^∗^*p* < 0.05, ^∗∗^*p* < 0.01 vs. the control group.

As shown in Figure 2A, stress exposure significantly reduced the percentage of sucrose consumption in stressed APP/PS1 mice in both the RIS and the RIS+ICA groups compared with the control animals [*F*(2,27) = 5.664, *p* = 0.009; *post hoc p* < 0.05 for both comparisons]. The results indicated that stress decreased sucrose preference, considered a major symptom of depression, in APP/PS1 mice.

#### Open Field Test

As shown in Figure 2B, the APP/PS1 mice in the two stress groups showed decreased horizontal and vertical movements compared with the control group [*F*(2,27) = 10.093, *p* = 0.001; *post hoc p* < 0.01 for both comparisons]. The results indicated that stress induced decreased interest in a new environment and behavioral suppression in APP/PS1 mice.

#### Elevated Plus Maze

As shown in Figure 2C, 4 weeks of exposure to RI stress did not significantly change the anxiety score of mice in the two stress groups [*F*(2,27) = 0.183, *p* = 0.834], indicating that the level of stress was too mild to induce anxiety-like behaviors.

#### Y Maze Test

As shown in Figure 2D, 4 weeks of exposure to RI stress did not significantly reduce the Ratio entry [*F*(2,27) = 0.101, *p* = 0.904], indicating the absence of memory impairment in stressed animals.

### Effect of Icariin on Learning and Memory in MWM

**Figure 3:**
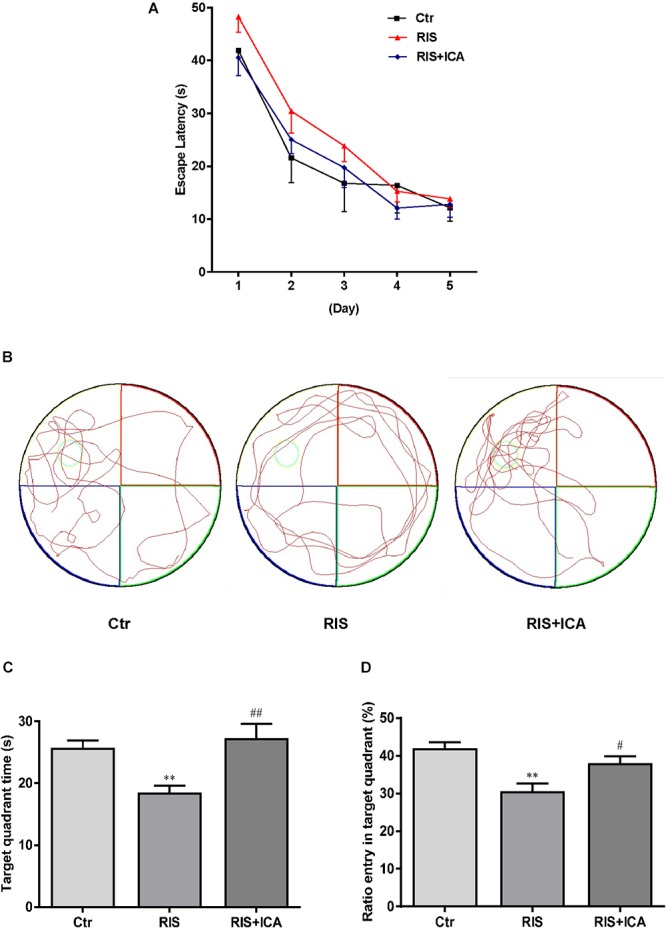
Effect of icariin on learning and memory in MWM. **(A)** The escape latency of training days. **(B)** The tracks in the MWM test. **(C)** Target quadrant time. **(D)** Ratio entry in the target quadrant. Mice in RIS group spent less time in the target quadrant and showed decreased entry ratio in the target quadrant in the MWM test, in comparison with the control group (*p* < 0.01). Icariin administration significantly relieved the behavioral alterations of the mice in the RIS+ICA group, compared with the RIS group. The results are expressed as the mean ± SEM, *n* = 10. ^∗∗^*p* < 0.01 vs. control group; ^#^*p* < 0.05, ^##^*p* < 0.01 vs. RIS group.

As shown in Figure 3A, mice in the RIS group had longer escape latency on the first, second, and third learning day, but there were no significant differences in escape latency at any day among the three groups [*F*(2,27) = 1.210, *p* = 0.314 (first day); *F*(2,27) = 0.902, *p* = 0.418 (second day); *F*(2,27) = 0.728, *p* = 0.492 (third day); *F*(2,27) = 0.419, *p* = 0.662 (fourth day) and *F*(2,27) = 0.146, *p* = 0.865 (fifth day)]. However, as shown in Figure 3B–D, mice in the RIS group spent less time in the target quadrant in the MWM test than the control group did [*F*(2,27) = 6.849, *p* = 0.004; *post hoc p* < 0.01]. Moreover, RIS mice showed decreased entry ratio in the target quadrant in comparison with the control mice [*F*(2,27) = 8.148, *p* = 0.002; *post hoc p* < 0.01]. Icariin administration significantly relieved these behavioral alterations in the RIS+ICA group as compared with the RIS group (*p* < 0.01, *p* < 0.05, respectively). The results indicated that long-term icariin administration effectively relieved the memory impairment of restraint/isolation-stressed mice.

### Effect of Icariin on Cytokines and NF-κB in the Hippocampus and PFC

#### Cytokines and NF-κB Expression in the Hippocampus

**Figure 4:**
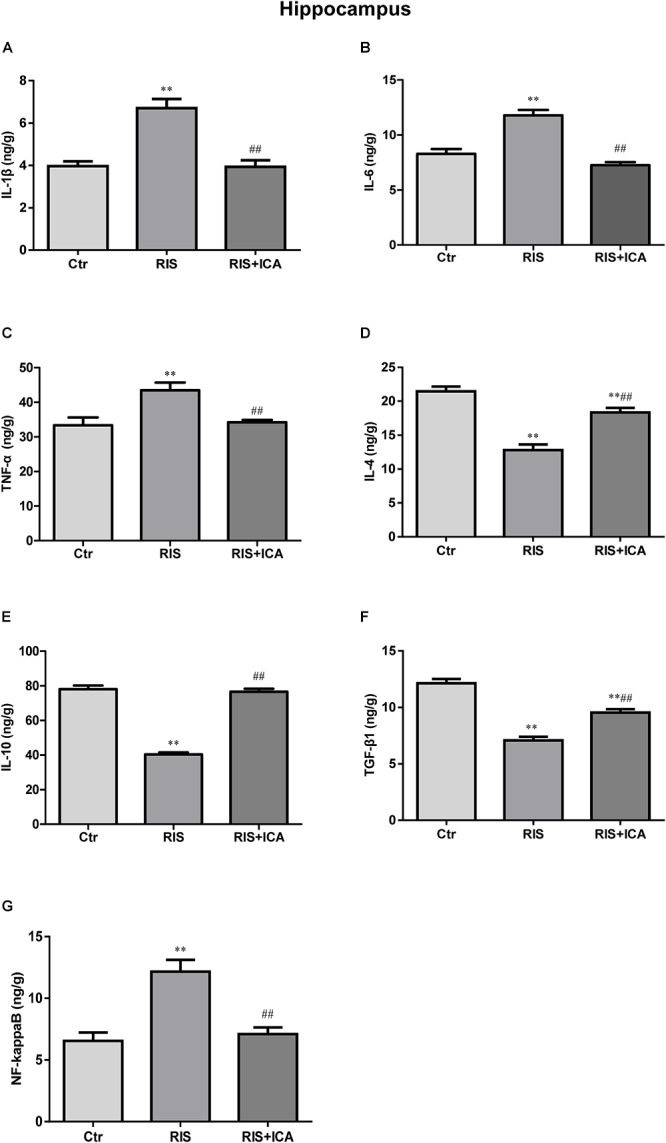
Effect of icariin on cytokines and NF-κB in the hippocampus. **(A–C)** The level of IL-1β **(A)**, IL-6 **(B)**, and TNF-α **(C)** increased in the RIS group, and icariin relieved the alteration. **(D–F)** The level of IL-4 **(D)**, IL-10 **(E)**, and TGF-β1 **(F)** decreased in the RIS group, and icariin relieved the alteration. **(G)** The level of NF-κB increased in the RIS group, and icariin relieved the alteration. The results are expressed as the mean ± SEM, *n* = 6. ^∗∗^*p* < 0.01 vs. control group; ^##^*p* < 0.01 vs. RIS group.

Cytokine expression in the hippocampus is shown in Figure 4A–F. Figure 4A shows that IL-1β levels increased in the RIS group compared with the control group [*F*(2,15) = 32.507, *p* < 0.001; *post hoc p* < 0.01]; however, icariin relieved the alteration (*p* < 0.01). Figure 4B shows that RIS significantly increased IL-6 levels [*F*(2,15) = 69.126, *p* < 0.001; *post hoc p* < 0.01] compared with controls, while icariin decreased them (*p* < 0.01). Figure 4C shows that stress increased TNF-α in APP/PS1 mice [*F*(2,15) = 10.718, *p* = 0.001, *post hoc p* < 0.01], while icariin could reverse this change (*p* < 0.01).

Figure 4D shows that IL-4 levels decreased in the RIS group compared with controls [*F*(2,15) = 74.971, *p* < 0.001; *post hoc p* < 0.01], and icariin relieved such alteration (*p* < 0.01). Similarly, Figure 4E shows that RIS significantly decreased IL-10 levels compared with controls [*F*(2,15) = 119.031, *p* < 0.001; *post hoc p* < 0.01], while icariin increased them (*p* < 0.01). Figure 4F shows that stress decreased TGF-β1 expression in APP/PS1 mice compared with controls [*F*(2,15) = 82.481, *p* < 0.001; *post hoc p* < 0.01], while icariin could reverse such change (*p* < 0.01).

Figure 4G shows that NF-κB levels increased in the RIS group compared with controls [*F*(2,15) = 16.709, *p* < 0.001: *post hoc p* < 0.01]; however, icariin relieved the alteration (*p* < 0.01).

#### Cytokines and NF-κB Expression in the PFC

**Figure 5:**
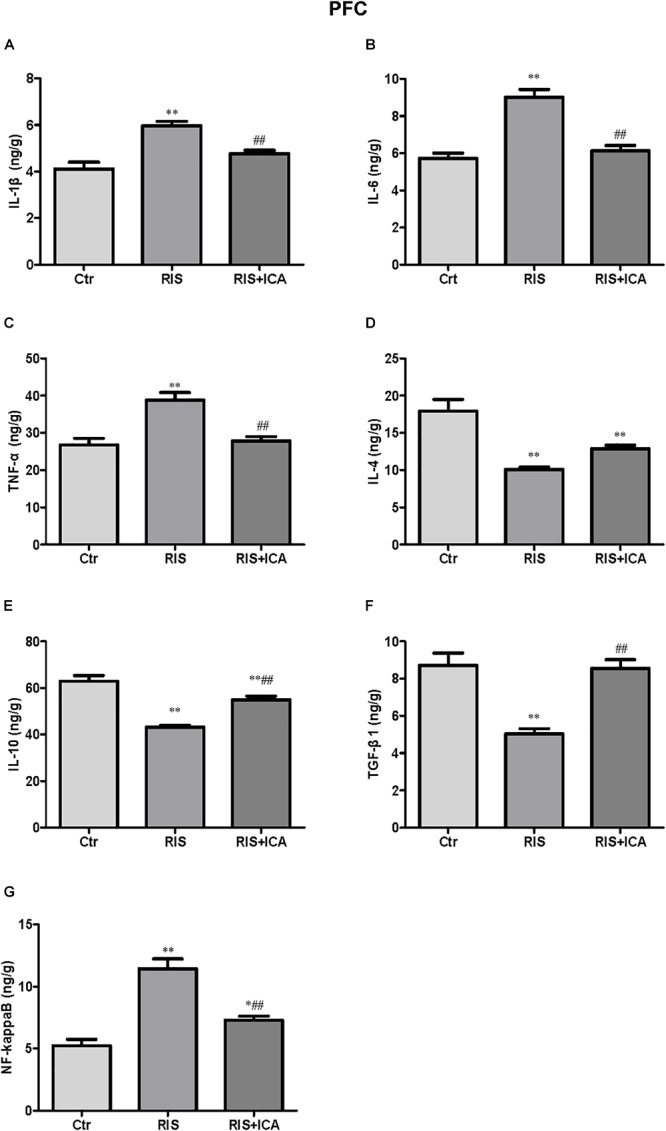
Effect of icariin on cytokines and NF-κB in the PFC. **(A–C)** The level of IL-1β **(A)**, IL-6 **(B)**, and TNF-α **(C)** increased in the RIS group, and icariin relieved the alteration. **(D)** The levels of IL-4 decreased in the RIS group, but icariin failed to relieve the alteration. **(E,F)** RIS significantly decreased the level of IL-10 **(E)** and TGF-β1 **(F)** compared with controls, and icariin could reverse the change. **(G)** NF-κB levels increased in the RIS group, and icariin relieved the alteration. The results are expressed as the mean ± SEM, *n* = 6. ^∗^*p* < 0.05, ^∗∗^*p* < 0.01 vs. control group; ^##^*p* < 0.01 vs. RIS group.

Cytokine expression in the PFC is shown in Figure 5A–F. Figure 5A shows that IL-1β levels increased in the RIS group compared with controls [*F*(2,15) = 17.565, *p* < 0.001; *post hoc p* < 0.01]; however, icariin relieved the alteration (*p* < 0.01). Figure 5B shows that RIS significantly increased the level of IL-6 [*F*(2,15) = 51.672, *p* < 0.001; *post hoc p* < 0.01], which was reversed by treatment of icariin (*p* < 0.01). Figure 5C shows that stress increased TNF-α expression compared with controls [*F*(2,15) = 16.475, *p* < 0.001; *post hoc p* < 0.01], while icariin could reverse this change (*p* < 0.01).

Figure 5D shows that IL-4 levels decreased in the RIS group compared with controls [*F*(2,15) = 25.263, *p* < 0.001; *post hoc p* < 0.01], and icariin failed to relieved this alteration. Figure 5E shows that RIS also significantly decreased the level of IL-10 [*F*(2,15) = 41.906, *p* < 0.001; *post hoc p* < 0.01], which was enhanced by the administration of icariin (*p* < 0.01). Figure 5F shows that stress decreased TGF-β1 compared with controls [*F*(2,15) = 23.709, *p* < 0.001; *post hoc p* < 0.01], while icariin could reverse this change (*p* < 0.01).

Figure 5G shows that the level of NF-κB increased in the RIS group compared with controls [*F*(2,15) = 37.217, *p* < 0.001; *post hoc p* < 0.01]; however, icariin relieved the alteration (*p* < 0.01).

### Effect of Icariin on Aβ Plaques and Microglia Phenotype in the Hippocampus and PFC

#### Aβ Plaques and Microglia Phenotype in the Hippocampus

**Figure 6:**
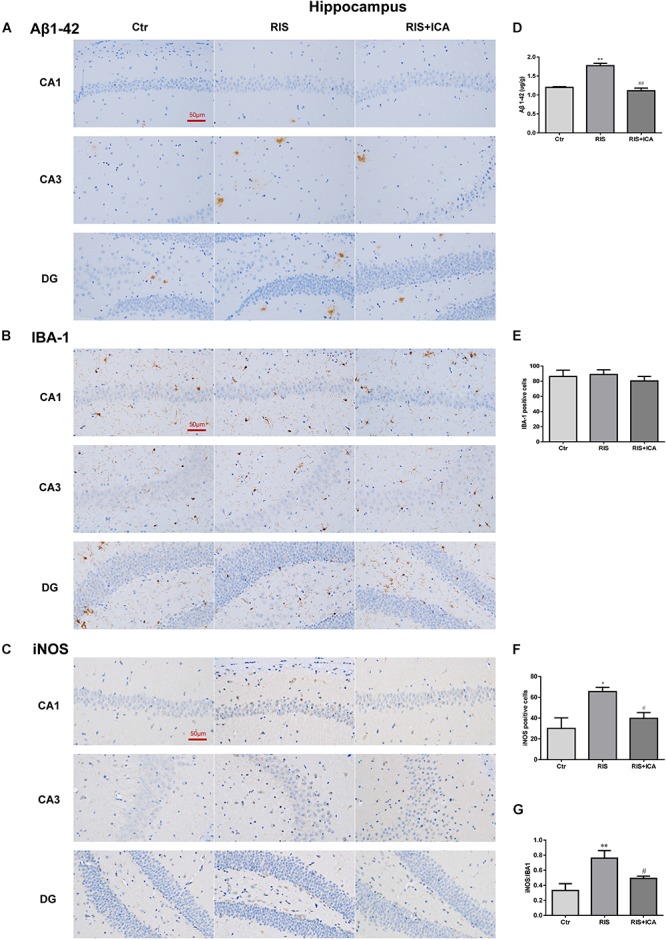
Effect of icariin on Aβ plaques and microglia phenotype in the hippocampus. **(A–C)** The immunohistochemical staining results of Aβ, Iba-1, and iNOS (400×). **(D)** RIS significantly increased the levels of Aβ in the RIS group compared with the controls, and administration of icariin decreased its levels in the RIS+ICA group. **(E)** The number of Iba-1+ microglia positive cells was not significantly different among the three groups. **(F)** The number of iNOS+ microglia positive cells in the hippocampus of stressed mice was higher than that of control mice, and icariin administration reversed the change in the RIS+ICA group. **(G)** The RIS group showed higher ratio of iNOS+ to Iba-1+ microglia, while the RIS+ICA group showed a lower ratio. The results of Aβ are expressed as the mean ± SEM, *n* = 6; the results of Iba-1 and iNOS are expressed as the mean ± SEM, *n* = 4. ^∗^*p* < 0.05, ^∗∗^*p* < 0.01 vs. control group; ^#^*p* < 0.05, ^##^*p* < 0.01 vs. RIS group.

As shown in Figure 6A, the amount and size of Aβ plaques in the hippocampus of the RIS APP/PS1 mice were larger than those in the mice of the control group, while icariin relieved the alteration in the RIS+ICA group. To accurately evaluate the changes in Aβ, we measured the Aβ level in the hippocampus using ELISA, as shown in Figure 6D. RIS significantly increased Aβ levels in the RIS group compared with the control group [*F*(2,15) = 74.140, *p* < 0.001; *post hoc p* < 0.01], while administration of icariin decreased the levels of Aβ in the RIS+ICA group (*p* < 0.01). The counting of Iba-1+ microglia positive cells did not reveal significant differences among the three groups as shown in Figure 6B,E [*F*(2,9) = 0.395, *p* = 0.685]. However, the number of iNOS+ microglia positive cells in the hippocampus of the stressed mice was higher than that in control mice [*F*(2,9) = 6.640, *p* = 0.017; *post hoc p* < 0.05], and icariin administration reversed the change in the RIS+ICA group (*p* < 0.05, Figure 6C,F). The higher ratio of iNOS+ microglia to Iba-1+ microglia in the stressed group and the lower ratio in the RIS+ICA group further support the promotion by icariin of a microglia phenotype switch in the hippocampus of RIS APP/PS1 mice [*F*(2,9) = 7.837, *p* = 0.011; *post hoc p* < 0.01, control vs. RIS; *p* < 0.05, RIS vs. RIS+ICA] (Figure 6G).

#### Aβ Plaques and Microglia Phenotype in the PFC

**Figure 7:**
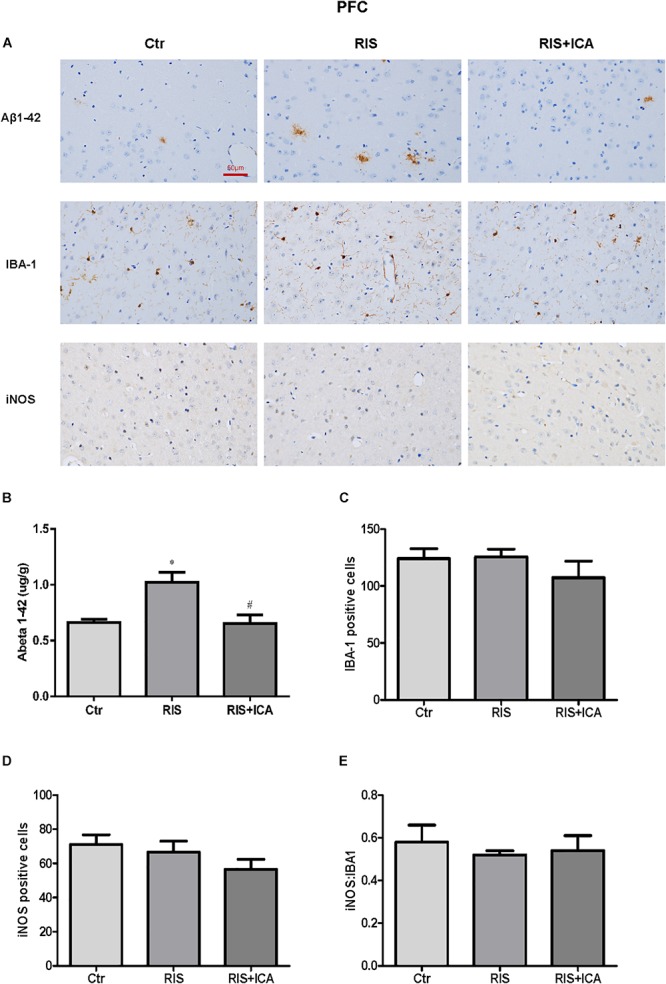
Effect of icariin on Aβ plaques and microglia phenotype in the PFC. **(A)** Results of immunohistochemical staining (400×). **(B)** RIS significantly increased the level of Aβ compared with controls, and icariin decreased it. **(C–E)** The number of Iba-1+ **(C)** and iNOS+ **(D)** microglia cells was not significantly different among the three groups. As a result, the ratio of iNOS+ to Iba-1+ microglia **(E)** was not significantly different either among the three groups. The results of Aβ are expressed as the mean ± SEM, *n* = 6; the results of Iba-1 and iNOS are expressed as the mean ± SEM, *n* = 4. ^∗^*p* < 0.05 vs. control group; ^#^*p* < 0.05 vs. RIS group.

As shown in Figure 7A, the amount and size of Aβ plaques in the PFC of RIS APP/PS1 mice were larger than that in the PFC of control mice, while icariin relieved such alterations. In order to accurately evaluate the change of Aβ, Aβ levels were also measured in the PFC using ELISA as shown in Figure 7B. Compared with controls, RIS significantly increased Aβ levels [*F*(2,15) = 4.368, *p* = 0.032; *post hoc p* < 0.05], while icariin decreased them (*p* < 0.05). The counting of Iba-1+ and iNOS+ microglia cells showed no significant differences among the three groups, as shown in Figure 7C,D [*F*(2,9) = 0.922, *p* = 0.432; *F*(2,9) = 1.502, *p* = 0.274, respectively]. As a result, the ratio of iNOS+ to Iba-1+ microglia also was not significantly different among the three groups [*F*(2,9) = 0.229, *p* = 0.800] (Figure 7E).

### Effect of Icariin on PPARγ in the Hippocampus and PFC

**Figure 8:**
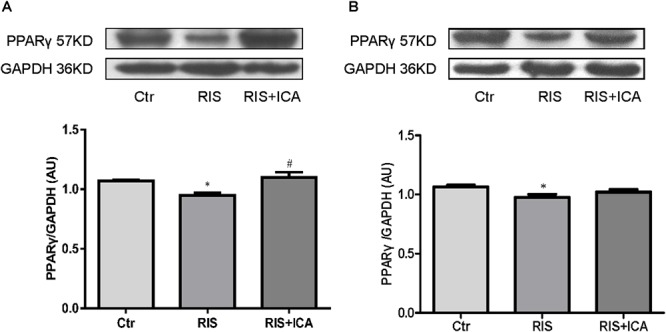
Effect of icariin on PPARγ in the hippocampus and PFC. **(A)** PPARγ expression in the hippocampus using western blotting. The RIS procedure induced a marked decrease in PPARγ levels in APP/PS1 mice compared with controls, and icariin could reverse the alteration. **(B)** PPARγ expression in the PFC using western blotting. The RIS procedure induced a marked decrease in PPARγ level in APP/PS1 mice compared with controls, and icariin could partially reverse the alteration. The results of hippocampus are expressed as the mean ± SEM, *n* = 4; the results of PFC are expressed as the mean ± SEM, *n* = 5. ^∗^*p* < 0.05 vs. control group; ^#^*p* < 0.05 vs. RIS group.

Figure 8A shows PPARγ expression in the hippocampus as assessed using western blotting. The RIS procedure induced a marked decrease in PPARγ levels in APP/PS1 mice compared with controls [*F*(2,9) = 7.917, *p* = 0.010; *post hoc p* < 0.05], and icariin could reverse such alteration (*p* < 0.05).

Figure 8B shows PPARγ expression in the PFC as assessed using western blotting. The RIS procedure induced a marked decrease in PPARγ levels in APP/PS1 mice compared with controls [*F*(2,12) = 3.916, *p* = 0.049; *post hoc p* < 0.05], and icariin could partially reverse the alteration (*p* > 0.05, control group vs. RIS+ICA).

## Discussion

Mounting evidence has shown that chronic psychosocial stress is a major risk factor for late-onset AD, and is associated with cognitive deficits (Piirainen et al., 2017). Chronic stress could prime the microglia and induce inflammatory responses in the adult brain, thereby compromising the synapse-supportive roles of microglia and contributing to age-related cognitive deterioration. In order to examine the effects of chronic stress on neuroinflammation and neurodegeneration in AD, we used restraint/isolation stress to build a stressed AD model (Jeong et al., 2006; Carroll et al., 2011; Lee and Han, 2013). Consistent with previous studies, 1 month of restraint/isolation stress induced lower sucrose consumption and lower horizontal and vertical movements in the OFT in APP/PS1 mice aged 4 months, compared with unstressed APP/PS1 mice, indicating that stressed animals showed anhedonia and were less interested in a new environment, thus suggesting the validity of the stressed AD model.

The role of chronic neuroinflammation in AD-related dementia has recently gained attention, implicating an early involvement of inflammation in the onset and progression of the disease (Querfurth and LaFerla, 2010). Microglia are the first line of defense against invading pathogens in the CNS (Piirainen et al., 2017). Studies have confirmed that stress could stimulate the microglia and prime them toward the M1 phenotype, which is responsible for neuroinflammation (Zhao et al., 2016; Tang et al., 2018). In the present study, M1 phenotype markers including IL-1β, IL-6, TNF-α, and NF-κB, and M2 markers such as IL-4, IL-10, and TGF-β1 in the hippocampus and the PFC were measured in 10-month-old mice in the control, RIS, and RIS+ICA groups by ELISA (Michelucci et al., 2009; Park et al., 2015). The results showed that stressed APP/PS1 mice had higher levels of M1 markers and lower levels of M2 markers in both the hippocampus and the PFC, indicating that stress induced microglia M1 activation in APP/PS1 mice. We also assessed the levels of the M1 phenotype marker iNOS and its ratio to the microglia marker Iba-1. We found that the fraction of M1 microglia was higher in the hippocampus, but not in the PFC of stressed mice than in control mice. A possible explanation is that the hippocampus is the area most sensitive to stress, because it shows the highest expression of corticosterone receptors among brain regions (Anacker et al., 2011; Zhang et al., 2018). Indeed the stress-induced activation of inflammatory pathways in the brain was shown to be region-specific (Ogundele et al., 2017). Given that the microglia M2 activation state was associated with the suppression of inflammation, promotion of phagocytosis and tissue repair (Chawla, 2010; Chinetti-Gbaguidi et al., 2011), as well as characterized by increased Aβ clearance and enhanced tissue remodeling (Heneka et al., 2013), increased microglia M1 activation in stressed APP/PS1 mice resulted in lower clearance ability and higher neurotoxicity of the microglia (Park et al., 2015). Moreover, microglia M1 activation and the release of inflammatory cytokines increased the neuroinflammation of brain, which could result in AD progression (Mandrekar-Colucci et al., 2012; Huang et al., 2017). In the present study, we observed that RIS mice had worse memory impairment in the MWM, together with higher levels of M1 phenotype markers and lower level of M2 phenotype markers in both hippocampus and PFC at 10 months of age, while icariin could attenuate these alterations. The results are consistent with the conclusion that the imbalance of M1/M2 microglia activation is involved in AD pathogenesis (Mandrekar-Colucci et al., 2012; Huang et al., 2017).

Previous studies confirmed that the failure of microglia to clear abnormally accumulating Aβ peptide led to neuroinflammation and neurodegeneration in AD models in which RIS elevated Aβ40 and Aβ42 levels in the brain, accelerated amyloid plaque formation, and impaired learning and memory (Jeong et al., 2006; Carroll et al., 2011; Lee and Han, 2013). In our study, the stressed APP/PS1 mice had higher level of Aβ accumulation in both the hippocampus and the PFC compared with unstressed mice. More importantly, the stressed APP/PS1 mice showed more severe memory impairment in the MWM test at 10 months of age compared with unstressed mice. These findings support the previous conclusion that psychological stress induces lower ability to clear accumulating Aβ peptide, resulting in impaired cognition (Jeong et al., 2006; Hoeijmakers et al., 2017).

The PPARs are a group of nuclear receptor proteins regulating gene expression as ligand-activated transcription factors (Michalik et al., 2006). Studies demonstrated that PPARγ can mediate the conversion of the microglia into the M2 phenotype (Yamanaka et al., 2012; Saijo et al., 2013). Chronic stress induced lower PPARγ expression in the adipose tissue and PPARγ knockout mice displayed more anxiety-like behaviors (Domi et al., 2016; Guo et al., 2017). Moreover, studies have suggested that activation of PPARγ-mediated anti-inflammatory signaling might be a potential therapeutic strategy for AD (Vallee et al., 2017). Our results showed that PPARγ expression levels in the hippocampus and the PFC were significantly lower in stressed than in unstressed mice, which indicates that stress induced microglia M1 activation is via suppression of PPARγ expression.

Epimedium (family Berberidaceae), commonly known as horny goat weed in the West, is known as Yin Yang Huo in Chinese medicine. Icariin is a highly potent active ingredient of this herb, believed to be the source of its many potential health benefits (Lee et al., 1995; Lin et al., 2004). Icariin was proven to possess anti-bacterial and anti-inflammatory efficacy (Luo et al., 2007; Zhou et al., 2011). A previous study indicated that it improved spatial learning and memory abilities in lipopolysaccharide (LPS)-induced rat brain dysfunction through the inhibition of hippocampus IL-1β and cyclooxygenase-2 (COX-2) expression (Guo et al., 2010). Accumulating evidence indicates that, in addition to inhibiting the activation of innate immune cells (including microglia) producing TNF-α and IL-1β, icariin led to stable suppression of NF-κB signaling activation in the hippocampus (Liu et al., 2015). Thus, icariin plays a counter-regulatory role resulting in neuroinflammation suppression. Studies have investigated the pharmacological properties of icariin in the CNS and showed that it can markedly attenuate cognitive deficits in several models of AD (Li et al., 2010; Nie et al., 2010; Jin et al., 2014), and also alleviate neuronal injury induced by ischemia (Li et al., 2005). It was also reported that icariin can attenuate inflammatory response through PPARγ activation in rats (Xiong et al., 2016). However, the role of icariin in attenuating PPARγ alterations in the AD brain has not yet been clarified. The present study compared the expression of PPARγ in the hippocampus and the PFC between controls, RIS, and RIS+ICA mice and found that stress induced lower PPARγ expression in the RIS mice, while icariin relieved such alteration in the RIS+ICA mice. Moreover, the results about the microglia activation phenotype and Aβ accumulation demonstrate that icariin administration normalized the Aβ clearance ability by up-regulating PPARγ in the RIS+ICA mice. As a result, the memory impairment of RIS+ICA mice was milder than that of untreated stressed mice.

## Conclusion

Our results demonstrate that RIS induced depressive-like behaviors, spatial memory impairment, over-expression of M1 microglia markers and increased Aβ accumulation by suppressing PPARγ in APP/PS1 mice. Icariin, which induces a microglia phenotype switch through the activation of PPARγ, is a promising candidate for new therapeutic strategies. In conclusion, our study may provide novel insights into the role of chronic psychosocial stress in the pathogenesis and progression of AD and the development of new therapeutic approaches. Icariin affecting the microglia phenotype and cytokine release through other pathways should be further studied both *in vitro* and *in vivo*.

## Data Availability

All datasets generated for this study are included in the manuscript and/or the supplementary files.

## Ethics Statement

All procedures used in the study were reviewed and approved by the Ethics Committee of School of Medicine, Shandong University, and comply with the National Institutes of Health’s Guide for the Care and Use of Laboratory Animals (NIH publication no. 85-23, revised 1985). In the handling and care of all animals, we followed the international guiding principles for animal research, as stipulated by the World Health Organization (WHO) Chronicle (World Health Organization, 1985), as adopted by the Laboratory Animal Center, Shandong University.

## Author Contributions

XL was involved in study design and data interpretation. YW performed the majority of the laboratory work and contributed to the analysis of data and the writing of the manuscript. TZ, MW, FZ, GZ, JZ, and YZ were responsible for the animal model and the behavioral tests. EW revised the manuscript. All authors approved the final version to be submitted.

## Conflict of Interest Statement

The authors declare that the research was conducted in the absence of any commercial or financial relationships that could be construed as a potential conflict of interest.
